# HMGA1 recruits CTIP2-repressed P-TEFb to the HIV-1 and cellular target promoters

**DOI:** 10.1093/nar/gku168

**Published:** 2014-03-11

**Authors:** Sebastian Eilebrecht, Valentin Le Douce, Raphael Riclet, Brice Targat, Houda Hallay, Benoît Van Driessche, Christian Schwartz, Gwenaëlle Robette, Carine Van Lint, Olivier Rohr, Arndt G. Benecke

**Affiliations:** ^1^Vaccine Research Institute, INSERM U955, Institut Mondor de Recherche Biomédicale, 8 rue du général Sarrail, 94011 Créteil, France, ^2^Institut des Hautes Études Scientifiques—Centre National de la Recherche Scientifique, 35 route de Chartres, 91440 Bures sur Yvette, France, ^3^Institut de Parasitologie et de Pathologie Tropicale, Fédération de Médecine Translationnelle, University of Strasbourg, 3 rue Koeberlé, 67000 Strasbourg, France, ^4^IUT Louis Pasteur, 1 Allée d’Athénes, 67300 Schiltigheim, France, ^5^Université Libre de Bruxelles (ULB), Service of Molecular Virology, Institute for Molecular Biology and Medicine (IBMM), 12 rue des Profs Jeener et Brachet, 6041 Gosselies, Belgium, ^6^Institut Universitaire de France—IUF, Paris, France and ^7^CNRS UMR 7224, Université Pierre et Marie Curie, 7 quai Saint Bernard, 75005 Paris, France

## Abstract

Active positive transcription elongation factor b (P-TEFb) is essential for cellular and human immunodeficiency virus type 1 (HIV-1) transcription elongation. CTIP2 represses P-TEFb activity in a complex containing 7SK RNA and HEXIM1. Recently, the inactive 7SK/P-TEFb small nuclear RNP (snRNP) has been detected at the HIV-1 core promoter as well as at the promoters of cellular genes, but a recruiting mechanism still remains unknown to date. Here we show global synergy between CTIP2 and the 7SK-binding chromatin master-regulator HMGA1 in terms of P-TEFb–dependent endogenous and HIV-1 gene expression regulation. While CTIP2 and HMGA1 concordingly repress the expression of cellular 7SK-dependent P-TEFb targets, the simultaneous knock-down of CTIP2 and HMGA1 also results in a boost in Tat-dependent and independent HIV-1 promoter activity. Chromatin immunoprecipitation experiments reveal a significant loss of CTIP2/7SK/P-TEFb snRNP recruitment to cellular gene promoters and the HIV-1 promoter on HMGA1 knock-down. Our findings not only provide insights into a recruiting mechanism for the inactive 7SK/P-TEFb snRNP, but may also contribute to a better understanding of viral latency.

## INTRODUCTION

In eukaryotic cells, RNA Polymerase II (RNA Pol II) is responsible for the transcription of protein-coding genes. On promoter clearance, the enzyme is paused in a promoter proximal position by the negative elongation factor and the 5,6-Dichloro-1-beta-D-ribofuranosylbenzimidazole (DRB) sensitivity-inducing factor (DSIF), which poises RNA Pol II to promptly react to upstream signals and generate full-length mRNA transcripts (1,2). The positive transcription elongation factor b (P-TEFb) is important to overcome this transcriptional block, as active P-TEFb, consisting of the Cyclin-dependent kinase 9 (Cdk9) as well as one of the Cyclins T1 (CycT1) or T2, phosphorylates negative elongation factor and DSIF as well as the carboxy-terminal domain of the paused polymerase, resulting in efficient transcriptional elongation (1–3). P-TEFb activity is negatively controlled in a ribonucleo-protein complex (RNP) containing the 7SK non-coding RNA and the Hexamethylene bis-acetamide inducible 1 (HEXIM1) protein (4,5). MePCE and LARP7 bind to the 5′- and the 3′-end of 7SK RNA, that way contributing to the maintenance of the integrity of this 7SK RNP (6,7). Besides the expression of cellular genes, also the expression of human immunodeficiency virus type 1 (HIV-1) essentially depends on P-TEFb, making HIV-1 a model system for studying P-TEFb function. Here, the viral transactivator of transcription (Tat) targets the inactive 7SK/P-TEFb RNP, displacing 7SK RNA and HEXIM1 to ensure efficient viral transcription (8,9). The 7SK/P-TEFb small nuclear RNP (snRNP) has recently been detected at the HIV-1 core promoter in a Sp1-dependent manner (8), where the Tat-recruited transcription factors PPM1G/PP2Cγ activate P-TEFb by mediating its release from the 7SK RNP (10), but a mechanism for 7SK RNP recruitment remains elusive to date.

In a recent study, we have identified the chicken ovalbumin upstream promoter transcription factor (COUP-TF) interacting protein 2 (CTIP2) as a novel compound of the 7SK/HEXIM1/P-TEFb snRNP, efficiently contributing to P-TEFb inactivation (11). CTIP2 is expressed in microglial cells, the resident macrophages of the central nervous system, which are the primary targets of productive HIV-1 infection within the brain (12). Moreover, high levels of CTIP2 expression in microglial cells from infected patients are correlated with HIV-1 post-integration latency (13). Besides the well-studied HIV-1–infected CD4+ T-lymphocytes they are considered as the major reservoir of latent virus (14). CTIP2 is an important factor for T-lymphocyte as well as spinal cord development (15,16) and has been shown to repress transcription of the integrated HIV-1 genome in microglial cells by several ways and thus might be a key factor for establishing and/or maintaining viral latency. In addition to its role as a negative regulator of P-TEFb, CTIP2 is able to repress HIV-1 gene expression by delocalizing Tat to the heterochromatin-associated protein HP1 (17). Furthermore, it can repress the initial phase of HIV-1 transcription by direct interaction with Sp1 and COUP-TF (18). CTIP2 promotes HIV-1 transcriptional silencing by recruiting the histone deacetylase 1 (HDAC1), HDAC2, the histone methyltransferase SUV39H1 as well as the histone demethylase Lysine (K)-specific demethylase 1 (LSD1) to the HIV promoter, which subsequently establish a heterochromatin environment (19–21).

The non-histone chromatin protein High mobility group AT-hook 1 (HMGA1) is able to interact with the loop2 substructure of 7SK RNA (22,23), which is also the interface between 7SK and CTIP2 (11). HMGA1 can associate with P-TEFb–bound 7SK RNA, and recent studies support a model of 7SK RNA-mediated recruitment of P-TEFb to the promoters of selected cellular target genes via HMGA1 (24). While we have recently identified HMGA1 to specifically interact with the HIV-1 transactivating response element (TAR), thereby repressing viral transcription likely by competing with cellular and viral activators (25), also the Sp1 binding site within the HIV-1 core promoter has been shown to be essential for efficient viral transcription (8). Besides the fact that HMGA1 interacts with a large variety of transcription factors, among those NF-κB and Sp1 (26), there are several high- and low-affinity binding sites for HMGA1 within the HIV-1 core promoter (27), pointing at additional roles of HMGA1 during HIV transcription.

Thus, we here set out to investigate whether HMGA1 is involved in the recruitment of CTIP2-inactivated P-TEFb to HIV-1 and cellular target promoters to regulate viral and endogenous gene expression.

## MATERIALS AND METHODS

### Cell culture and transfections

HEK293, human microglial cells [CMEH3 cell line, corresponding to clone 3 described by Janabi *et al.* (28)] and HIV-latently infected CHME-5 cells (29) were cultured as described previously (11). Transfections were performed using FugeneHD (Roche) or JetPrime (Polyplus) according to the manufacturer’s instructions. The plasmids used for 7SK knock-down (24), 7SK overexpression (23), dnCdk9 expression (24), HMGA1 knock-down/overexpression (22,24) and CTIP2 knock-down/overexpression (19) have been described previously. Cell viability was tested 48 h after transfection by measuring the mitochondrial dehydrogenase activity using the Water soluble tetrazolium (WST)-1 reduction assay (Roche) following manufacturer’s instructions. The viability of cells transfected with control short hairpin (sh)RNA was arbitrarily set to 100%.

### Immunoprecipitation assays and western blot analyses

Immunoprecipitations of HMGA1-FLAG fusions were performed from nuclear extracts of transiently transfected HEK293 cells using M2 anti-FLAG agarose (Sigma) as recommended by the manufacturer. Endogenous HMGA1 was immunopurified from nuclear extracts using a specific anti-HMGA1 antibody (Abcam). Immunopurifications with boiled anti-HMGA1 IgG were used as a control. Sodium dodecyl sulphate-polyacrylamide gel electrophoresis (SDS-PAGE) and blotting were performed as described previously (22). Antibodies used in western blot analyses were M2 anti-FLAG antibody (Sigma), anti-HMGA1 antibody (Abcam), anti-CTIP2 antibody (Abcam), anti-Cdk9 antibody (Pierce), anti-CycT1 antibody (Proteintech), anti-HEXIM1 antibody (Proteintech) and anti-LARP7 antibody (Proteintech) with the corresponding secondary horseradish peroxidase-coupled antibodies (Sigma) as recommended by the manufacturer.

### Quantitative reverse transcriptase-polymerase chain reaction

Quantitative reverse transcriptase-polymerase chain reaction (qRT-PCR) analyses were performed as described previously (22,30). Primers to amplify 7SK RNA were 5′-CAT CCC CGA TAG AGG AGG AC-3′ (sense) and 5′-GCC TCA TTT GGA TGT GTC TG-3′ (antisense). U6 RNA primers were 5′-CGC TTC GGC AGC ACA TAT AC-3′ (sense) and 5′-AAA ATA TGG AAC GCT TCA CGA-3′ (antisense). HIV transcripts were detected with primers directed against the TAR region: 5′-GTT AGA CCA GAT CTG AGC CT-3′ (sense) and 5′-GTG GGT TCC CTA GTT AGC CA-3′ (antisense). β-Actin mRNA primers were 5′-GTC GAC AAC GGC TCC GGC-3′ (sense) and 5′-GGT GTG GTG CCA GAT TTT CT-3′ (antisense).

### HIV-1 promoter-driven reporter assays

On shRNA-mediated knock-down or overexpression of HMGA1 and/or CTIP2, CMEH3 microglial cells were transfected with a HIV-1 Long terminal repeat (LTR)-driven luciferase reporter in the presence or in the absence of HIV-1 Tat-FLAG. The efficiencies of the used constructs have been previously validated (19,20). After 48 h, cells were harvested and luciferase activity was quantified using the Dual Luciferase® Reporter Assay System and GloMax®-96 Microplate Luminometer (Promega) according to the manufacturers’ instructions. A renilla reporter was used for normalization. To assess the relative viral expression, a HIV-1 LTR-driven reporter containing the full viral genome fused to the luciferase reporter was used.

For transcription assays with integrated virus, HIV-latently infected CHME-5 cells (29) were transfected with CTIP2- (19) and/or HMGA1-targeting shRNA vectors (22) in comparison with a non-targeting shRNA vector as a control (22) and HIV gene expression was quantified 48 h after transfection by quantitative RT-PCR (29).

### Chromatin immunoprecipitation assays

Chromatin immunoprecipitation (ChIP) assays were performed as described previously (31). HEK293 cells were transfected with the HIV-1 LTR-driven luciferase reporter, FLAG-CTIP2, HMGA1-targeting shRNA or non-targeting control shRNA constructs. pcDNA3 vector (Invitrogen) has been used as control. ChIP assays were performed 48 h after transfection according to the Millipore protocol. Cells were cross-linked with 1% formaldehyde for 10 min at 37°C. After quenching of formaldehyde with 125 mM glycine for 5 min at room temperature, the fixed cells were washed with 1× PBS, harvested and centrifuged at 1300 rpm at 4°C for 5 min. The cell pellet was resuspended in lysis buffer (0.1% SDS, 50 mM HEPES, pH 7.9, 140 mM NaCl, 1 mM EDTA, 1% Triton X-100, 0.1% Na-deoxycholate) to a final concentration of 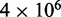 cells/400 μl in presence of protease inhibitors (1 mM phenylmethylsulfonyl fluoride (PMSF), 1× protease inhibitor coctail (PIC)), incubated on ice for 10 min and sonicated with a Bioruptor sonicator (Diagenode, Philadephia, PA, USA; 30 s on–30 s off cycles for 30 min at high intensity). The solubilized chromatin was clarified by centrifugation for 10 min at 13 000 rpm at 4°C and the supernatant was diluted 5-fold in ChIP dilution buffer (16.7 mM Tris–HCl at pH 8.1, 1.2 mM EDTA, 167 mM NaCl, 0.01% SDS and 1.1% Triton X-100) for immunoprecipitation. The primary antibodies used for ChIP were anti-CTIP2 (bethyl), anti-Cdk9 (Santa Cruz), anti-Cyclin T1 (Santa Cruz) and anti-Hexim-1 (Abcam). Immunocomplexes were collected by rotation for 2 h at 4°C with 50 μl of protein A sepharose preblocked with yeast tRNA and BSA. The beads were washed sequentially for 4 min at 4°C with rotation, once each with low salt buffer (20 mM Tris–HCl at pH 8.1, 2 mM EDTA, 150 mM NaCl, 0.1% SDS and 1% Triton X-100), high salt buffer (20 mM Tris–HCl at pH 8.1, 2 mM EDTA, 500 mM NaCl, 0.1% SDS and 1% Triton X-100), LiCl buffer (10 mM Tris–HCl at pH 8.1, 1 mM EDTA, 0.25 M LiCl, 1% NP40 and 1% Deoxycholate) and twice with Tris-EDTA (TE) buffer (10 mM Tris–HCl at pH 8.0, 1 mM EDTA). Immunocomplexes were eluted twice with 250 μl of elution buffer (1% SDS, 0.1 M NaHCO_3_) for 15 min at room temperature. Eluates and input chromatin were heated to 65°C overnight in the presence of 0.2 M NaCl to reverse formaldehyde cross-linking, treated with 2 μl of proteinase K (10 mg/ml), 10 μl of 0.5 M EDTA and 20 μl of 1 M Tris–HCl (pH 6.5) for 1 h at 45°C. DNA was phenol/chloroform extracted and precipitated with ethanol plus glycogen as carrier. Immunoprecipitated DNA was subjected to real-time PCR quantification on an ABI Prism 7000 Ligthcycler using the Power SYBR Green kit (Applied Biosystems). The specificity of the enrichment has been controlled by amplification of the GAPDH gene. The following primer sequences were used: HIV-1 promoter: 5′-CAG CTG CTT TTG CCT GTA CTG-3′ (forward) and 5′-TCC ACA CTG ACT AAA AGG GTC TGA-3′ (reverse); luciferase gene: 5′-CCG TGA TGG AAT GGA ACA AC-3′ (forward) and 5′-CAT AGA ACT GCC TGC GTC A-3′ (reverse); Epithelial stromal interaction 1 (EPSTI1) promoter: 5′-CGT GGC TGA TCC CAG TTA TT-3′ (forward) and 5′-CCA GAG AAC TTC CCC ACG TA-3′ (reverse); Poly [ADP-ribose] polymerase 12 (PARP12) promoter: 5′-TAC CTC CCA GGT AGG GTT CC-3′ (forward) and 5′-GCC CTT TGA GGA GGT AAT CC-3′ (reverse).

### Gene expression profiling and data analysis

Total RNA from transfected microglial cells was prepared using the RNeasy Midi Kit (Qiagen) as recommended by the manufacturer. The RNA was labelled using the Illumina® TotalPrep^TM^ RNA Amplification Kit (Ambion) according to the manufacturer’s instructions. For microarray analyses, hybridization and detection were performed following the protocols supplied by Illumina using the HumanHT-12 v4 Expression BeadChip Kit and an iScan system (both Illumina). The raw data were quality controlled (32), NeoNORM normalized using k = 0.2 (33) and analyzed as outlined in (34,35). For subtraction profiling we used the CDS statistical test (36) with a positive false discovery rate correction where appropriate. Canonical pathway enrichment studies were performed using Ingenuity Pathway Analysis® software (Ingenuity® Systems) as recommended by the manufacturer. Transcriptome data were deposited in the public database MACE (http://mace.ihes.fr) using Accession Nos.: 3037572262 (CTIP2 knock-down), 2166467750 (CTIP2 overexpression), 3164892326 (HMGA1 knock-down), 2365090982 (HMGA1 overexpression), 2401152166 (7SK knock-down), 3197764774 (7SK overexpression) and 2967448742 (dnCdk9 expression).

## RESULTS

### HMGA1 interacts with CTIP2 in an RNA-independent fashion

HMGA1 interacts with the 7SK/P-TEFb snRNP via the loop2 substructure of the RNA (22–24), while CTIP2, besides interacting with the same part of 7SK, is also able to directly bind HEXIM1 in an RNA-independent manner (11). Thus, we tested first, whether HMGA1 and CTIP2 can interact and whether 7SK RNA can mediate an interaction between HMGA1, CTIP2 and P-TEFb.

**Figure 1.**
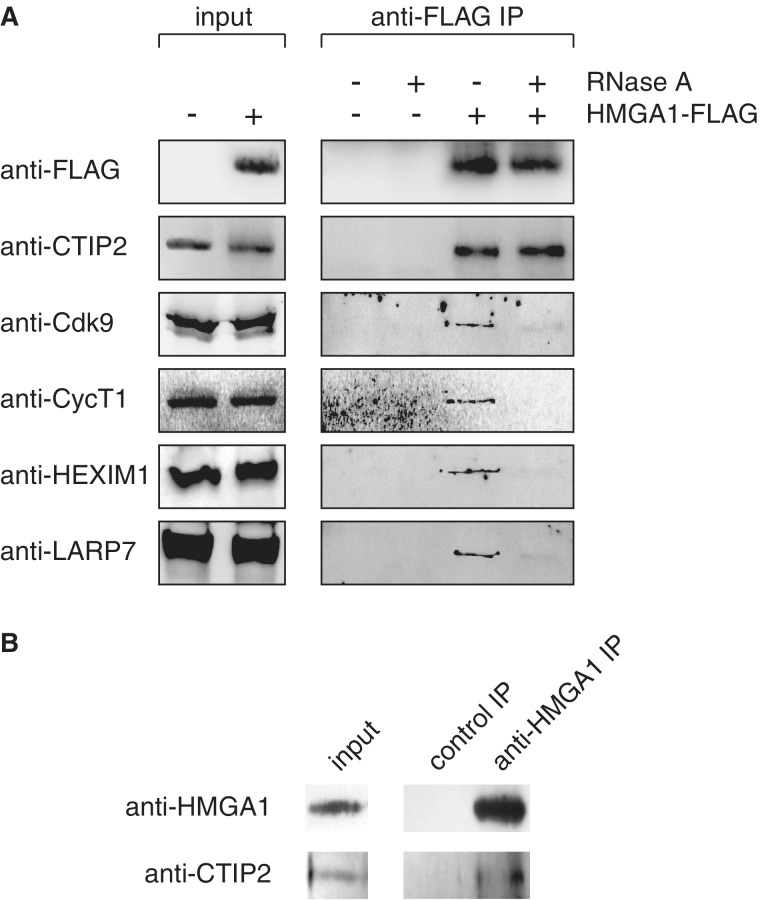
HMGA1 interacts with CTIP2 in an RNA-independent fashion, while the association with inactive P-TEFb is RNase-sensitive. (**A**) Immunopurification of HMGA1-FLAG from transiently transfected HEK293 cells in the presence or the absence of RNase A. Input and precipitation steps were monitored for endogenous CTIP2, Cdk9, CycT1, HEXIM1 and the 7SK-associated LARP7 in western blot analyses. Mock-transfected cells were used as a control. (**B**) Immunopurification of endogenous HMGA1 from microglial cells using a specific anti-HMGA1 antibody. Input and precipitation steps were monitored for endogenous HMGA1 and CTIP2. An immunopurification with boiled anti-HMGA1 IgG was used as a control.

For that we performed immunopurifications of HMGA1-FLAG from transiently transfected HEK293 cells in the presence and absence of RNase A and subsequently monitored the amounts of co-purified endogenous CTIP2 (Figure 1A). A significant amount of CTIP2 co-precipitates with HMGA1 in an RNase-resistant fashion, while a portion of the inactive P-TEFb snRNP, namely, Cdk9, CycT1 and HEXIM1 as well as the stably 7SK-associated protein LARP7 associates with HMGA1 via RNA (Figure 1A). As CTIP2 interacts with HMGA1 in an RNA-independent manner, it adds to a number of previously reported HMGA1-interacting partners, such as AP1, NF-κB, c-Jun, PU.1 or Sp1 (26,37–39), suggesting that HMGA1 might be involved in the recruitment of CTIP2 and CTIP2-associated enzymes. To verify the association of the endogenously expressed proteins, we performed an immunopurification of endogenous HMGA1 from microglial cells using a specific anti-HMGA1 antibody and monitored the precipitation step for endogenous CTIP2 (Figure 1B). Also, endogenous CTIP2 co-purified with endogenous HMGA1.

### HMGA1 and CTIP2 cooperatively repress P-TEFb–dependent gene expression

Our hitherto results allow for a functional connection of HMGA1 and CTIP2, which might affect the expression of P-TEFb target genes. To test this hypothesis, we used global gene expression profiling to identify and compare CTIP2, HMGA1 and P-TEFb target genes in microglial cells.

**Figure 2.**
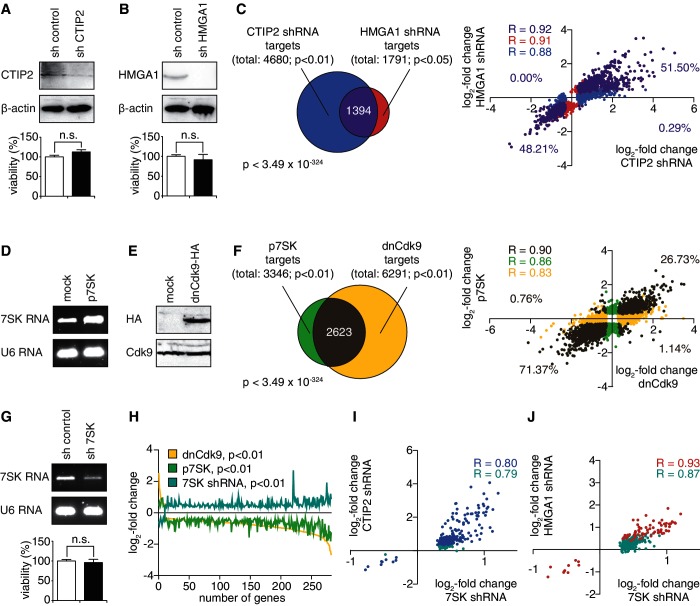
HMGA1 and CTIP2 repress the expression of 7SK-dependent P-TEFb target genes. (**A**) shRNA-mediated knock-down of CTIP2 in microglial cells. CTIP2 levels were monitored in western blot analyses and β-actin was used as a control (upper panel). Cell viability on shRNA-mediated knock-down was measured by MTT test (lower panel). n.s., not statistically significant in a students *t*-test. (**B**) shRNA-mediated knock-down of HMGA1 in microglial cells. HMGA1 amounts were quantified by western blot analyses, and β-actin was used as a housekeeping gene (upper panel). Cell viability was measured as in (A) (lower panel). (**C**) Venn diagram (left panel) and scatter plot (right panel) of the CTIP2-target genes (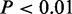, blue), the HMGA1-target genes (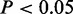, red) and the common subset (purple). The gene numbers of each set, the hypergeometric distribution *P*-value as well as the Pearson correlation coefficients for all three subsets and the fractions located in each quadrant of the scatter plot are indicated. (**D**) Overexpression of 7SK RNA in microglial cells. RNA amounts were monitored by ethidium bromide staining on agarose gel electrophoresis, and U6 RNA was used as a control. (**E**) Overexpression of a HA-tagged, dominant negative (dn) version of Cdk9 in microglial cells. dnCdk9 expression was monitored in western blot analyses using anti-HA and anti-Cdk9 antibodies. (**F**) as in (C), but for the target genes of dnCdk9 (yellow) and 7SK RNA overexpression (green). (**G**) shRNA-mediated knock-down of 7SK RNA in microglial cells. RNA amounts were monitored as in (D). Cell viability was measured as in (A). (**H**) Identification of 7SK-dependent P-TEFb targets as genes, whose expression is concomitantly regulated on dnCdk9 expression (yellow), 7SK RNA overexpression (green) and 7SK RNA knock-down (turquoise). The number of genes is plotted on the x-axis and the log_2_-fold change in each condition is plotted on the y-axis. Genes are plotted in descending order based on their expression changes on dnCdk9 expression. (**I**) Scatter plot comparing the expression changes of the P-TEFb targets identified in (H) on 7SK knock-down with their expression changes on CTIP2 knock-down. Genes statistically significantly regulated on CTIP2 knock-down are coloured in blue and the Pearson correlation coefficient is indicated. (**J**) Scatter plot comparing the expression changes of the P-TEFb targets identified in (H) on 7SK knock-down with their expression changes on HMGA1 knock-down. Genes statistically significantly regulated on HMGA1 knock-down are coloured in red.

To identify genes, whose expression is affected by CTIP2 and HMGA1, the expression of endogenous CTIP2 and HMGA1 was knocked down using specific shRNAs (Figure 2A and B), before gene expression changes were assessed using microarray analyses. On CTIP2 knock-down, 4680 genes were regulated, of which 3525 genes (75%) were up-regulated and 1155 (25%) down-regulated (Supplementary Figure S1A). Thus, CTIP2 predominantly represses target gene expression, which is in line with previously reported roles of CTIP2 (8,9). The HMGA1 knock-down resulted in the regulation of 1791 genes, of which 782 genes (44%) were up- and 1009 genes (56%) were down-regulated (Supplementary Figure S1A). Comparing the HMGA1 and the CTIP2 target genes, we observe a common subset of 1394 genes being significantly regulated in both conditions (Figure 2C, left panel). The expression of 1391 genes (99.71%) among the common subset is highly positively correlated (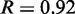) when comparing CTIP2 knock-down and HMGA1 knock-down (Figure 2C, right panel). Including the full target sets of CTIP2 or HMGA1 does not affect the strong positive correlation (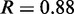 and 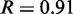, respectively), uncovering that CTIP2 and HMGA1 affect gene expression in the same manner and that the CTIP2 and HMGA1 target genes are virtually identical in microglial cells (Figure 2C, right panel). To identify 7SK-dependent P-TEFb target genes, we performed transcriptome profiling of microglial cells on 7SK RNA overexpression (Figure 2D) and overexpression of a dominant negative (dn), kinase-dead version of Cdk9 fused to a HA-tag (Figure 2E). Because 7SK RNA is a negative regulator of P-TEFb, its overexpression is expected to shift the equilibrium of P-TEFb towards its inactive state as it is the case for the overexpression of dnCdk9. 7SK RNA overexpression led to a differential expression of 3346 genes, of which 962 genes (29%) showed an elevated expression and 2384 genes (71%) exhibited a diminished expression (Supplementary Figure S1A). The expression of dnCdk9 resulted in the differential expression of 6291 genes, among those, 2667 genes (42%) being up- and 3624 genes (58%) being down-regulated (Supplementary Figure S1A). The target genes of 7SK overexpression and dnCdk9 expression show a statistically highly significant (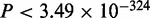) common subset of 2623 genes (Figure 2F, left panel), whose expression changes are highly positively correlated (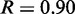) when comparing 7SK overexpression and dnCdk9 expression (Figure 2F, right panel). Notably, the expression of 1872 genes (71% of the common subset) is down-regulated in both conditions, which is in concordance with the role of 7SK RNA as a negative P-TEFb regulator. However, to work on a reliable set of 7SK target genes, we included a condition of shRNA-mediated 7SK knock-down (Figure 2G). Ideally, P-TEFb target genes, which are down-regulated by the overexpression of 7SK RNA, should be up-regulated by a 7SK RNA knock-down. Such a knock-down led to a statistically significant (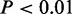) regulation of 3826 genes, of which 3354 genes (88%) were up- and 472 genes (12%) were down-regulated (Supplementary Figure S1A). We defined those genes, whose expression was concordingly affected by 7SK overexpression, 7SK knock-down and dnCdk9 expression in a statistically significant manner (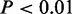), meaning, for example, genes whose expression was down-regulated by 7SK and dnCdk9 overexpression and up-regulated by 7SK knock-down, as 7SK-dependent P-TEFb target genes (Figure 2H). Among those, 264 genes (96%) are activated and 10 genes (4%) are repressed by P-TEFb, which is in line with the general gene-activating function of P-TEFb. We performed canonical pathway enrichment studies to identify those cellular pathways, which are affected most in each single condition, revealing that various pathways are affected in several conditions simultaneously (Supplementary Figure S1B–E).

**Figure 3.**
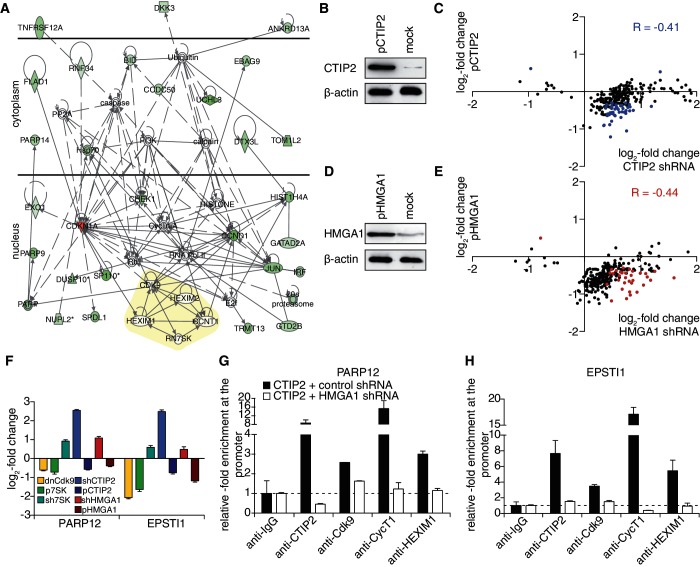
HMGA1 recruits CTIP2-inactivated P-TEFb to cellular target promoters. (**A**) Network of 7SK-dependent P-TEFb target genes affected by both knock-down of CTIP2 and HMGA1. Up-regulated genes are coloured in red, down-regulated genes are coloured in green. Functional connections are indicated as arrows. The inactive P-TEFb snRNP is highlighted in yellow (Cdk9, CycT1, HEXIM1, 7SK RNA). (**B**) The overexpression of CTIP2 (pCTIP2) was verified by western blot analyses and β-actin was used as a housekeeping gene. (**C**) Scatter plot comparing the expression changes of 7SK-dependent P-TEFb targets on CTIP2 knock-down and overexpression. Genes statistically significantly (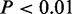) regulated in both conditions are highlighted in blue. (**D**) The overexpression of HMGA1 (pHMGA1) was verified by western blot analyses, and β-actin was used as a housekeeping gene. (**E**) As in (C), but comparing HMGA1 knock-down and overexpression. Genes statistically significantly (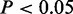) regulated in both conditions are highlighted in red. (**F**) Representative visualization of the effects of dnCdk9 expression, 7SK overexpression, 7SK knock-down, CTIP2 knock-down, CTIP2 overexpression, HMGA1 knock-down and HMGA1 overexpression on the expression of the 7SK-dependent P-TEFb targets PARP12 and EPSTI1. (**G**) ChIP analyses targeting the EPSTI1 promoter from HEK293 cells transfected with CTIP2-FLAG in the presence or absence of a HMGA1 knock-down. (**H**) Same as in (G), but for the PARP12 promoter.

To assess the role of CTIP2 and HMGA1 during the expression of these 7SK-dependent P-TEFb target genes, we compared their expression changes on 7SK knock-down with their differential expression on CTIP2 (Figure 2I) and HMGA1 knock-down (Figure 2J). In both cases the gene expression changes are highly positively correlated (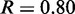 for CTIP2 and 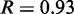 for HMGA1), showing that both CTIP2 and HMGA1 concomitantly repress the expression of 7SK-dependent P-TEFb target genes. Network analyses based on these genes place the inactive P-TEFb snRNP upstream of our observed target genes, confirming our experimental approach (Figure 3A). To confirm that our observations were due to a repressive effect of CTIP2 and HMGA1 on 7SK-dependent P-TEFb target genes, we added global gene expression analyses of CTIP2 and HMGA1 overexpressing microglial cells (Figure 3B and D). The majority of the 7SK-dependent P-TEFb targets, whose expression was enhanced on CTIP2 and HMGA1 knock-down, was repressed on CTIP2 and HMGA1 overexpression (Figure 3C and E).

### HMGA1 is involved in the recruitment of CTIP2 and CTIP2-repressed P-TEFb to cellular gene promoters

Our findings support a model in which HMGA1 is involved in the recruitment of the P-TEFb repressor CTIP2 and/or the CTIP2-repressed P-TEFb snRNP to target promoters, as HMGA1 interacts with a large variety of transcription factors and also with specific DNA target sites.

To answer this question, we randomly chose PARP12 and EPSTI1 as two 7SK-dependent P-TEFb target genes, whose expression is repressed by CTIP2 as well as HMGA1 (Figure 3F). We performed ChIP analyses from HEK293 cells overexpressing CTIP2 to investigate the impact of CTIP2 levels on the recruitment of the P-TEFb snRNP to the gene promoters (Figure 3G and H). The overexpressed CTIP2 itself located at the PARP12 and the EPSTI1 promoter and resulted in an increased recruitment of the inactive P-TEFb snRNP—Cdk9, CycT1 and HEXIM1 (Figure 3G and H, black bars). A simultaneous knock-down of HMGA1 led to a significant loss of the recruitment of CTIP2 and the inactive P-TEFb snRNP (Figure 3G and H, white bars), supporting a role of HMGA1 during the recruitment of CTIP2-inactivated P-TEFb.

### HMGA1 and CTIP2 synergistically repress HIV-1 promoter activity

HIV-1 gene expression critically depends on P-TEFb function. Thus, we set out to investigate the impact of CTIP2 and HMGA1 on HIV-1 transcription.

**Figure 4.**
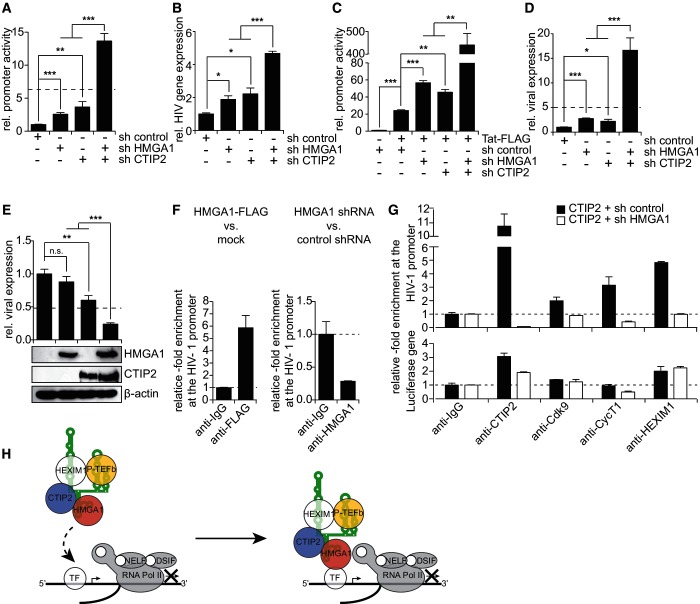
HMGA1 and CTIP2 cooperatively repress HIV-1 gene expression by a HMGA1-mediated recruitment of CTIP2-inactivated P-TEFb to the HIV promoter. (**A**) HIV-1 LTR-driven reporter assays in microglial cells on CTIP2 and/or HMGA1 knock-down. The magnitude of an additive effect of CTIP2 and HMGA1 knock-down is indicated as a dotted line. *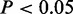, **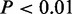, ***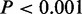 in a Student’s t-test. (**B**) Transcription assays in HIV-latently infected CHME-5 cells on CTIP2 and/or HMGA1 knock-down. HIV gene expression was quantified by qRT-PCR and normalized to β-actin expression as a housekeeping gene. HIV gene expression on expression of a non-targeting control shRNA was arbitrarily set to 1. *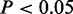, ***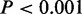 in a Student’s t-test. (**C**) As in (A), but in microglial cells expressing HIV-1 Tat-FLAG. (**D**) As in (A), but using the pNL4.3 delta ENV-luciferase proviral plasmid as reporter construct. (**E**) As in (D), but in microglial cells overexpressing CTIP2-FLAG and/or HMGA1-FLAG. Protein overexpression was verified in western blot experiments using anti-FLAG antibody. β-actin was used as a housekeeping gene. (**F**) ChIP analyses targeting the HIV-1 promoter from HEK293 cells on expression of HMGA1-FLAG (left panel) or knock-down of endogenous HMGA1 (right panel). (**G**) ChIP analyses targeting the HIV-1 promoter from HEK293 cells transfected with CTIP2-FLAG in the presence or absence of a HMGA1 knock-down (upper panel). The luciferase gene was used as a control (lower panel). (**H**) Model: The CTIP2-repressed 7SK/P-TEFb snRNP is recruited to cellular and viral promoters by the interaction of 7SK L2-bound HMGA1 with core promoter-bound basal transcription factors (TF, e.g. NF-κB or Sp1) or DNA.

We performed HIV-1 promoter-driven luciferase reporter assays in microglial cells on shRNA-mediated knock-down of HMGA1 and/or CTIP2 both in the presence and in the absence of Tat (Figure 4). In the latter case, the HMGA1 knock-down alone results in an increase of HIV-1 promoter activity, which is comparable with that on CTIP2 knock-down (Figure 4A). The simultaneous knock-down of both proteins results in a boost of promoter activity, which goes beyond an additional effect of each single knock-down, indicating a synergistic pathway being involved (Figure 4A). To confirm our findings, which were obtained using a non-integrated reporter plasmid, in the context of an integrated provirus, we knocked down CTIP2, HMGA1 or both proteins simultaneously in HIV-latently infected CHME-5 cells (29) and subsequently measured viral transcription by qRT-PCR (Figure 4B). Also in the context of a latently integrated provirus, a knock-down of CTIP2 and HMGA1 results in a statistically significant induction of viral transcription, which is boosted in a synergistic manner when both proteins are knocked down simultaneously (Figure 4B). Also in the presence of Tat the knock-down of HMGA1 or CTIP2 leads to an increased HIV-1 promoter activity, which is boosted when both proteins are knocked down simultaneously (Figure 4C). Next, we quantified HIV-1 expression on modulations of CTIP2 and HMGA1 levels in microglial cells. As expected, the concomitant knock-downs of CTIP2 and HMGA1 synergistically stimulate the expression of the pNL4.3 delta envelope (ENV) luciferase provirus, in which the ENV gene has been replaced by the luciferase reporter (Figure 4D). Consistent with these findings, the overexpression of both proteins collaborates in silencing HIV-1 expression (Figure 4E).

### HMGA1 recruits CTIP2-inactivated P-TEFb to the HIV-1 promoter

As it is the case for host cellular P-TEFb target genes, also the repressive synergistic cooperativity of CTIP2 and HMGA1 in the case of HIV-1 transcription might be a consequence of a HMGA1-mediated recruitment of the CTIP2-inactivated 7SK/P-TEFb snRNP to the HIV promoter.

HMGA1 localizes at the HIV-1 core promoter, as verified by ChIP experiments from HEK293 cells on overexpression of HMGA1-FLAG or knock-down of endogenous HMGA1 (Figure 4F). To investigate the impact of CTIP2 levels on the recruitment of the P-TEFb snRNP to the HIV promoter, we performed ChIP analyses from HEK293 cells carrying the HIV-1 promoter-driven luciferase reporter construct and overexpressing CTIP2 (Figure 4G). As it was the case for the host cellular genes PARP12 and EPSTI1, the overexpression of CTIP2 increased the recruitment of the inactive P-TEFb snRNP to the HIV-1 core promoter (Figure 4G). Also in the case of the HIV-1 promoter, the simultaneous knock-down of HMGA1 led to a significant loss of the recruitment of CTIP2 and the inactive P-TEFb snRNP (Figure 4G, upper panel). The luciferase gene downstream of the HIV-1 promoter showed neither a significant enrichment of the inactive P-TEFb snRNP nor a significant impact of the HMGA1 knock-down (Figure 4G, lower panel). Together, these findings support a model, in which HMGA1 not only recruits the CTIP2-repressed P-TEFb snRNP to cellular gene promoters, but also to the HIV-1 core promoter.

## DISCUSSION

We here establish a global cooperativity of HMGA1 and CTIP2 in terms of gene expression regulation, including the regulation of 7SK-dependent P-TEFb target genes. While CTIP2 contributes to Cdk9 inactivation as a part of the inactive 7SK/P-TEFb snRNP in microglial cells (11), HMGA1 has been previously proposed to be involved in the recruitment of P-TEFb to selected target promoters (24). The inactive 7SK/P-TEFb snRNP has been detected at the HIV-1 core promoter in a Sp1-dependent fashion (8). A recent study has provided evidence for the 7SK/P-TEFb snRNP also associating with active endogenous gene promoters in a genome-wide manner (40). This study suggests an involvement of the serine arginine-rich (SR) protein SRSF2 (SC35) in the recruitment of activated P-TEFb to paused RNA Pol II via an interaction with the nascent transcript, a mechanism similar to the Tat/TAR interaction in the case of HIV. Our findings suggest that HMGA1 is important for the repressive function of CTIP2 and support a model in which HMGA1 mediates the recruitment of CTIP2-inactivated P-TEFb to cellular and viral gene promoters, where SRSF2 or HIV-1 Tat are subsequently needed for P-TEFb activation (Figure 4H). Notably, HMGA1 has been previously found to co-localize with SC35 at hypoxic conditions, which might provide plasticity between the recruitment of inactive P-TEFb to the promoter and P-TEFb activation under stress conditions (41). However, the strong global synergy between HMGA1 and CTIP2 may go beyond a cooperative P-TEFb pathway and may include, for instance, also a HMGA1-mediated recruitment of CTIP2 and CTIP2-associated chromatin-modifying enzymes. Also, the recruitment of the CTIP2-binding histone deacetylases HDAC1, HDAC2 and the histone demethylase LSD1 have been shown to depend on Sp1 (19,20). Because we have shown the latter complex to be distinct from the inactive CTIP2/7SK/P-TEFb snRNP (11), both recruitment pathways would likely be mutually exclusive.

Taken together, the HMGA1-mediated recruitment of CTIP2-repressed P-TEFb not only affects endogenous gene expression in microglial cells but, moreover, also modulates the expression of the HIV-1 genome. The identification of host-cellular factors involved in HIV-1 silencing, as accomplished here, contributes to a more detailed understanding of how viral latency is established and maintained and thus may open ways to novel therapeutic approaches for fighting HIV infection (42,43,44).

## SUPPLEMENTARY DATA

Supplementary Data are available at NAR Online.

## FUNDING

ANRS (to O.R., B.T. and A.B.); Sidaction, Institut Universitaire de France (to O.R.); the Genopole Evry; the Institut des Hautes Études Scientifiques; and the Centre National de la Recherche Scientifique (CNRS) (to A.B.). C.V.L. is *Directeur de Recherches* of the Belgian Fund for Scientific Research (FRS-FNRS, Belgium). Work in C.V.L.’s lab was supported by grants from the FRS-FNRS (Belgium), the Télévie-Programme of the FRS-FNRS, the Programme *d’Excellence Cibles* of the Walloon region, the NEAT (European AIDS treatment network) integration grant, the International Brachet Stiftung, the *Fondation Roi Baudouin* (Belgium) and the ANRS. Funding for open access charge: Centre National de la Recherche Scientifique.

*Conflict of interest statement*. None declared.

## Supplementary Material

Supplementary Data
