# Supplementary material for: HMGA1 recruits CTIP2-repressed P-TEFb to the HIV-1 and cellular target promoters
Source: Nucleic Acids Res. 2014 Mar 11;42(8):4962–71. doi: 10.1093/nar/gku168 (PMC4005653; doi:10.1093/nar/gku168)
Supplement: Supplementary Data [file supp_42_8_4962__index.html]

HMGA1 recruits CTIP2-repressed P-TEFb to the HIV-1 and cellular target promoters — HMGA1 recruits CTIP2-repressed P-TEFb to the HIV-1 and cellular target promoters — Supplementary Data 

# HMGA1 recruits CTIP2-repressed P-TEFb to the HIV-1 and cellular target promoters

## Supplementary Data

files

**Files in this Data Supplement:**

- Supplementary Data - zip file
