# Supplementary material for: HMGA1 recruits CTIP2-repressed P-TEFb to the HIV-1 and cellular target promoters
Source: Nucleic Acids Res. 2014 Mar 11;42(8):4962–71. doi: 10.1093/nar/gku168 (PMC4005653; doi:10.1093/nar/gku168)

Supplemental figure legends

Figure S1: Identification of CTIP2-, HMGA1-, 7SK- and Cdk9-target genes in microglial cells. (A) Transcriptome heatmaps of genes significantly ($p<<0.05$) differentially expressed upon shRNA-mediated knock-down of CTIP2, HMGA1 or 7SK RNA or overexpression of 7SK RNA or dnCdk9 in microglial cells. Non-targeting control shRNA expressing or mock transfected cells have been used as control. L indicates the $\log_2$-fold change in expression, S indicates the signal, P indicates the p-value and the roman numbers indicate independent biological replicates. (B) The canonocal pathway enrichment for the CTIP2-target genes was calculated using Ingenuity Pathway Analysis software. The 22 most significantly enriched pathways are displayed. The threshold p-value (0.05) is shown as a dotted line. The ratio corresponds to the number of genes significantly regulated in the given condition divided by the total number of genes involved in the corresponding pathway. (C) as in (B), but for the HMGA1-target genes. (D) and as in (B), but for the combined 7SK-target genes. (E) as in (B), but for the Cdk9-target genes.

Eilebrecht et al., 2014, Figure S1

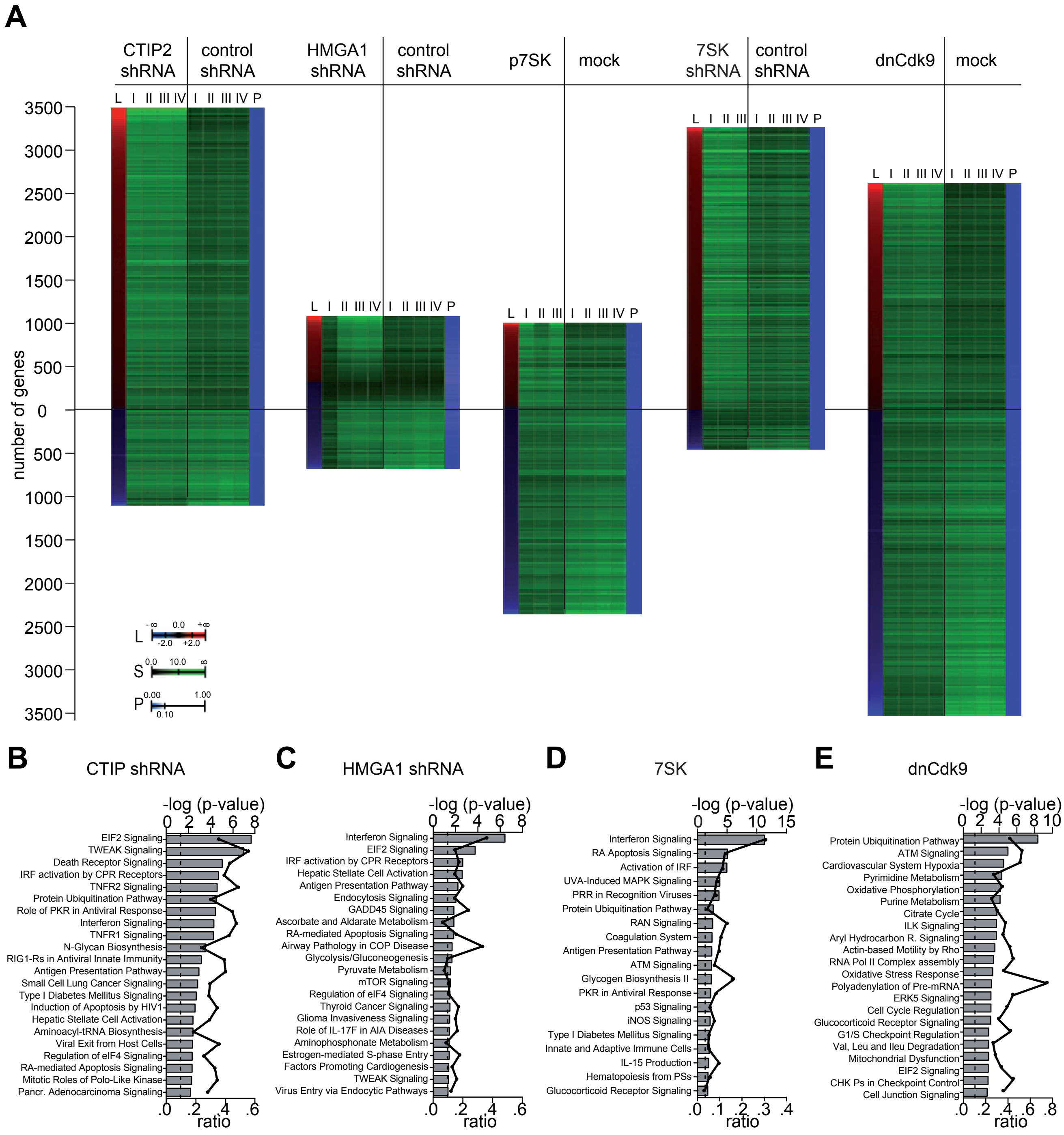

Supplement: Supplementary Data [file supp_gku168_supplemental_data.zip › supplemental_data_eilebrecht_2014.pdf.7zkhngs.partial]
